# *In utero *exposure to a low concentration of diesel exhaust affects spontaneous locomotor activity and monoaminergic system in male mice

**DOI:** 10.1186/1743-8977-7-7

**Published:** 2010-03-23

**Authors:** Tomoharu Suzuki, Shigeru Oshio, Mari Iwata, Hisayo Saburi, Takashi Odagiri, Tadashi Udagawa, Isamu Sugawara, Masakazu Umezawa, Ken Takeda

**Affiliations:** 1 Department of Hygiene Chemistry, Faculty of Pharmaceutical Sciences, Tokyo University of Science, 2641 Yamazaki, Noda-city, Chiba 278-8510, Japan; 2 Department of Hygiene Chemistry, School of Pharmaceutical Sciences, Ohu University, 31-3 Misumido, Tomita-cho, Koriyama-city, Fukishima 963-8611, Japan; 3 Department of Molecular Pathology, Research Institute of Tuberculosis, 3-1-24 Matsuyama, Kiyose-city, Tokyo 204-8533, Japan; 4 Core Research for Evolutional Science and Technology (CREST), Japan Science and Technology Agency), 4-1-8 Hon-cho, Kawaguchi-city, Saitama 332-0012, Japan; 5 School of Medicine and Medical Sciences, University of Tsukuba, 1-1-1 Tennodai, Tsukuba-city, Ibaraki 305-8577, Japan

## Abstract

**Background:**

Epidemiological studies have suggested that suspended particulate matter (SPM) causes detrimental health effects such as respiratory and cardiovascular diseases, and that diesel exhaust particles from automobiles is a major contributor to SPM. It has been reported that neonatal and adult exposure to diesel exhaust damages the central nervous system (CNS) and induces behavioral alteration. Recently, we have focused on the effects of prenatal exposure to diesel exhaust on the CNS. In this study, we examined the effects of prenatal exposure to low concentration of diesel exhaust on behaviour and the monoaminergic neuron system. Spontaneous locomotor activity (SLA) and monoamine levels in the CNS were assessed.

**Methods:**

Mice were exposed prenatally to a low concentration of diesel exhaust (171 μg DEP/m^3^) for 8 hours/day on gestational days 2-16. SLA was assessed for 3 days in 4-week-old mice by analysis of the release of temperature-associated infrared rays. At 5 weeks of age, the mice were sacrificed and the brains were used for analysis by high-performance liquid chromatography (HPLC).

**Results and Discussion:**

Mice exposed to a low concentration of diesel exhaust showed decreased SLA in the first 60 minutes of exposure. Over the entire test period, the mice exposed prenatally to diesel exhaust showed decreased daily SLA compared to that in control mice, and the SLA in each 3 hour period was decreased when the lights were turned on. Neurotransmitter levels, including dopamine and noradrenaline, were increased in the prefrontal cortex (PFC) in the exposure group compared to the control group. The metabolites of dopamine and noradrenaline also increased in the PFC. Neurotransmitter turnover, an index of neuronal activity, of dopamine and noradrenaline was decreased in various regions of the CNS, including the striatum, in the exposure group. The serum corticosterone level was not different between groups. The data suggest that decreased SLA in mice exposed prenatally to diesel exhaust is due to facilitated release of dopamine in the PFC.

**Conclusions:**

These results indicate that exposure of mice *in utero *to a low concentration of diesel exhaust decreases SLA and alters the neurochemical monoamine metabolism of several regions of the brain.

## Background

Several epidemiological studies have shown a positive association between the level of ambient particulate matter (PM) and mortality caused by respiratory and cardiovascular diseases [1,2]. Diesel engines produce large amounts of PM, and the health effects of exposure to diesel exhaust have been studied. According to several reports, diesel exhaust and diesel exhaust particles (DEPs), the particulate components of diesel exhaust, can affect the central nervous system (CNS). An epidemiological study showed a group of railroad workers exposed to diesel exhaust had impairment of neurobehaviour [3]. Subsequent studies showed that severe air pollution is associated with brain inflammation, Alzheimer's-like pathology [4-6], disruption of blood-brain barrier [6] and cognitive deficit [7,8]. The exposure of human volunteers to ambient levels of DEPs showed abnormal electrical signals of their frontal cortex, the important area for higher brain functions [9]. Animal models have been used to clarify the mechanisms underlying the effects of diesel exhaust exposure. The molecular toxicity of diesel exhaust is suggested to include oxidative stress-mediated inflammation, which is considered to be central to both pulmonary and systemic adverse health effects [10,11]. It was reported that nano-sized DEPs selectively damage cultured dopaminergic neurons by oxidative insult [12] and intracranial microinjection of fractionated diesel exhaust in rat hippocampus and striatum induces tissue damage in these regions [13]. Furthermore, in a recent study, DEPs induced oxidative stress and increased NF-κB at the blood-brain barrier of mice [14]. Another study showed that intranasal administration of nano-sized carbon black particles modulated the increase of expression of inflammatory cytokine mRNA, such as interleukin-1β, and the levels of amino acid neurotransmitters, such as glutamate and glycine, in the mice olfactory bulb induced by lipoteichoic acid [15]. Regarding the effects of prenatal exposure to diesel exhaust (0.3 - 3.0 mg DEP/m^3^), numerous caspase-3-positive cells were found in the cerebral cortex and hippocampus of newborn mice [16]. A subsequent study showed that prenatal exposure to diesel exhaust (1.0 mg DEP/m^3^) decreased the dopamine turnover, an index of dopamine neuronal activity, in the striatum [17]. However, there are no data to show the effects of prenatal exposure to low concentration of diesel exhaust on the CNS. The present study showed alteration of spontaneous locomotor activity (SLA) and monoamine levels in the CNS of mice following *in utero *exposure to a low concentration of diesel exhaust (0.171 mg DEP/m^3^) that corresponds to 1.71-fold of the Japanese environmental quality standard of daily averaged level of suspended particulate matter (SPM).

## Methods

### Animals

Twenty-six pregnant ICR mice were purchased from Japan SLC Inc. (Shizuoka, Japan) and housed under controlled conditions with 12 hours light/12 hours dark cycle and *ad libitum *access to food and water. They were divided into two groups: diesel exhaust exposure group (*n *= 12) and control group (*n *= 14). The mice of the exposure group were exposed to diesel exhaust for 8 hours/day (9:00 - 17:00 h.), for 5 days per week (Monday-Friday) in an inhalation chamber at the Research Institute of Tuberculosis (Japan Anti-Tuberculosis Association, Tokyo, Japan) from gestational days (GD) 2 - 16. After the exposure period, mothers and pups were maintained in a clean room. Pregnant mice delivered their pups on GD 19. The number and the sex ratio of pups in the exposure group and the control group were 114 (male:female = 75:39) and 161 (75:86), respectively. On postnatal day (PND) 4, the number of pups per litter was adjusted randomly to ten. In each group, pups were weaned on PND 21, after which male mice were transported to Tokyo University of Science (Chiba, Japan). Mice were transported carefully to minimize stress factors by Sankyo Labo Service Co., Inc. (Tokyo, Japan) and Tokyo Laboratory Animals Science Co., Ltd. (Tokyo, Japan). All experimental animals were handled in accordance with institutional and national guidelines for the care and use of laboratory animals.

### Exposure to diesel exhaust

A 2369 cc diesel engine (Isuzu Motors Ltd., Tokyo, Japan) was operated at a speed of 1050 rpm and at 80% load with a commercial oil. The exhaust was introduced into a stainless steel dilution tunnel (450 mm diameter × 6250 mm), where the exhaust was mixed with clean air, and average concentrations of exhaust constituents were maintained at 1.06 × 10^4 ^suspended particles/cm^3 ^(171 μg/m^3^), 1.25 ppm for carbon monoxide (CO), 0.04 ppm for nitrogen dioxide (NO_2_), and less than 0.01 ppm for sulfur dioxide (SO_2_).

### Behavioural analysis

The SLA of each mouse was measured in a transparent acrylic cage (20 cm × 31 cm × 13 cm) with an activity monitor with an infrared ray sensor (NS-AS01; Neuroscience Inc., Tokyo, Japan). The analysis was done when the mice were 4 weeks old (*n *= 10/group). Movement was measured according to the release of temperature-associated infrared rays. SLA counts were collected at 10 min intervals for 3 days. Data were analyzed automatically with a computerized system (multidigital 32-port counter system; Neuroscience Inc.). The analysis was conducted in "a new environment" and in "a home cage environment", which refers to the first 60 min test period just after moving into a new cage and the subsequent test period each day, respectively. Statistical analysis was done with two-way, repeated-measures analysis of variance (ANOVA), in which the variables were diesel exhaust exposure and time, followed by post hoc Student's *t*-test. The level of statistical significance was set at *P *< 0.05.

### Sampling procedure

Following the behavioural test, brain and trunk blood were obtained from the animals (5 weeks of age). The body weight of the animals was 28.56 - 37.51 g and there was no significant difference in body weight between the exposure group (32.7 ± 2.4 g) and the control group (32.9 ± 2.0 g). The brain was dissected into six regions, immediately frozen in liquid nitrogen, and stored at -80°C. Serum was separated in a gel barrier capillary blood collection tube (Capiject T-MG; Terumo Medical Corp., Elkton, MD) followed by centrifugation at 2200 *g *at 4°C for 15 min and stored at -80°C until analysis.

### Brain dissection

Brain dissection was done according to the modified method of Heffner *et al*. [18] and was based on the atlas described by Paxinos and Franklin [19]. The following four regions were dissected from frozen forebrain and midbrain coronal sections on a silicon plate chilled with dry ice: prefrontal cortex (PFC; containing cingulated cortex and motor cortex areas 1 and 2); striatum (dorsal); hippocampus (caudal) and midbrain (containing ventral tegmental area and substantia nigra). Determination of monoamine levels was done in PFC, striatum, hippocampus, midbrain, cerebellum, and brainstem.

### Preparation of homogenates

Frozen brain tissues was homogenized in ice-cold 0.2 M perchloric acid (Nacalai Tesque Inc., Kyoto, Japan) containing 100 μM Na_2_-EDTA (Dojinto Laboratories, Kumamoto, Japan) and 1 ng/mL isoproterenol as an internal standard (Sigma-Aldrich Co., St. Louis, MO). The homogenates were kept on ice for 30 min and centrifuged at 20,000 *g *at 0°C for 15 min. The supernatant was mixed with 1 M sodium acetate to adjust the pH to 3.0 (Kanto Chemical Co., Inc., Tokyo, Japan) and were frozen immediately in liquid nitrogen and stored at -80°C. The precipitate was used for the protein assay.

### High-performance liquid chromatography (HPLC)

Each group contained samples from 10 mice. A 10 mL sample of the final supernatant was injected with a microsyringe (702SNR; Hamilton Co., Reno, NV) into an HPLC system equipped with an electrochemical detector (HTEC-500MAB; Eicom Co., Kyoto, Japan). The standard solution contained the monoamines dopamine and noradrenaline and their metabolites. The dopamine metabolites were 3-methoxytyramine hydrochloride (3-MT), 3,4-dihydroxyphenylacetic acid (DOPAC) and homovanillic acid (HVA). The noradrenaline metabolites were normetanephrine hydrochloride (NM) and 4-hydroxy-3-methoxyphenylglycol hemipiperazinium (MHPG). Standards dopamine, HVA, 3-MT, NM and MHPG were obtained from Sigma-Aldrich. Standards noradrenaline and DOPAC were obtained from Nacalai Tesque and Wako Pure Chemical Industries, Ltd. (Osaka, Japan), respectively. Separation of monoamines and their metabolites was done by passage through a C18 reverse-phase column (Eicompak SC-5ODS; 3.0 mm × 150 mm; Eicom), maintained at 25°C and connected to an electrochemical detector (EPC-500, Eicom). The mobile phase was 0.1 M acetic acid/citric acid buffer (pH 3.5) containing Na_2_-EDTA (5 mg/L), octanesulfonic acid (190 mg/L; Nacalai Tesque), and methanol (15% (v/v); Kanto Chemical Co., Inc.). The flow rate was maintained at 0.5 mL/min for 35 min. Data were collected and analysed with the PowerChrom 280 System (eDAQ Pty Ltd., New South Wales, Australia). To determine the protein concentration, pellets were dissolved in 100 mM Tris-HCl for protein determination by a high-sensitivity version of the Bradford method with a commercial reagent (ADV-01; Cytoskeleton Inc., Denver, CO), and measurements were done according to the manufacturer's protocol. The absorbance at 595 nm was measured with a 96-well microplate reader (model 550; Bio-Rad Laboratories Inc., Hercules, CA), and protein concentration was calculated from a standard curve generated with bovine γ-globulin (Pre-Diluted Protein Assay Standards: Bovine Gamma Globulin Set; Thermo Fisher Scientific Inc., Rockford, IL). Concentrations of monoamines and their metabolites are expressed as pg mg^-1 ^of protein, and the catabolism rate is expressed as the ratio of metabolite to monoamine (e.g. HVA/dopamine). Indices were calculated from individual tissue samples. Statistical analysis was done with the Mann Whitney *U*-test. The level of statistical significance was set at *P *< 0.05.

### Measurement of serum corticosterone

The concentration of corticosterone in serum was determined with a Correlate-EIA Corticosterone Enzyme Immunoassay Kit (Assay Designs Inc., Ann Arbor, MI).

## Results

### Spontaneous locomotor activity (SLA)

**Figure 1:**
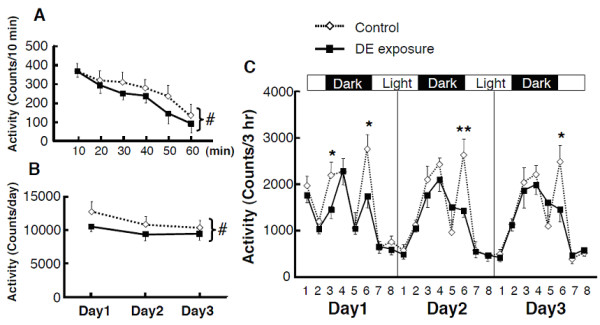
**Effect of prenatal exposure to diesel exhaust on spontaneous locomotor activity (SLA) in mice**. Each group contained 10 mice. (A) During the initial 60 minutes acclimating to the novel environment, the mice exposed prenatally to diesel exhaust showed lower SLA than the control group. (B) Throughout the testing period, the mice of the exposure group showed lower daily SLA than those of the control group. (C) SLA in each 3 hour period was altered in diesel exhaust-exposed mice. Post hoc analysis revealed that SLA was decreased at some time points. All data are presented as mean ± SEM. The level of statistical significance was set at * *P *< 0.05.

SLA was measured continuously for 3 days. During the first 60 min, a decrease in locomotor activity in the mice exposed prenatally to diesel exhaust was found by two-way, repeated-measures ANOVA (Figure 1A). A decrease in daily locomotor activity over the entire testing period was also found (Figure 1B). SLA in each 3 hour period was also altered in the exposure group, and post hoc analysis showed that SLA was decreased at some time points (Figure 1C). During the first day, datapoints 3 and 6 showed significantly decreased values. During the second and third days, datapoint 6 showed decreased values.

### The levels of monoamine and their metabolites

**Figure 2:**
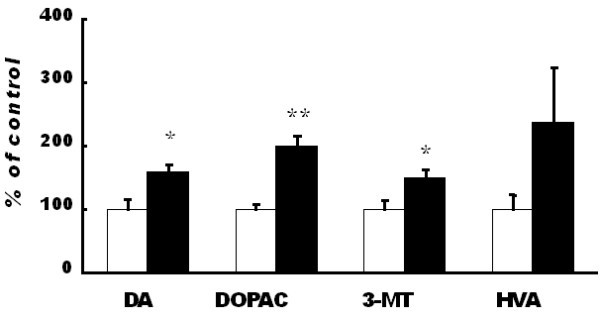
**Dopamine and its metabolites in the prefrontal cortex**. Dopamine, DOPAC and 3-MT were increased significantly in the exposure group compared to those in the control group. The dopamine turnover (DOPAC/dopamine, 3-MT/dopamine, HVA/dopamine A and [DOPAC + HVA]/dopamine) were not different between the groups. Data are presented as mean ± SEM. The level of statistical significance was set at * *P *< 0.05.

**Figure 3:**
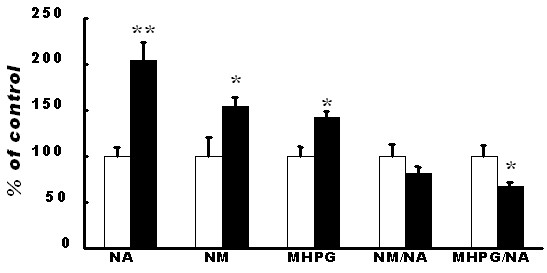
**Noradrenaline, its metabolites and turnover in the prefrontal cortex**. Noradrenaline, NM and MHPG were increased significantly in the exposure group compared to those in the control group. The noradrenaline turnover (MHPG/noradrenaline) was decreased compared to that in the control group. Data are presented as mean ± SEM. The level of statistical significance was set at * *P *< 0.05.

The determination of monoamine levels was conducted in six brain regions: PFC, striatum, hippocampus, midbrain, cerebellum and brainstem. In PFC, the levels of dopamine and noradrenaline were increased in the exposure group (Figure 2, 3). The dopamine level in brainstem was decreased (Table 1) but the dopamine and noradrenaline levels were not altered in the other regions. The levels of metabolites of dopamine were increased in the PFC (Figure 2), and the level of HVA was increased in the brainstem (Table 1). These metabolites were decreased in the hippocampus and in the midbrain (Table 1). The levels of metabolites of noradrenaline were increased in the PFC (Figure 3) but were decreased in the other regions (Table 2). With respect to neurotransmitter turnover, an index of neuronal activity calculated as a ratio of metabolite to transmitter was decreased in some regions of the brain. The turnover of dopamine was decreased in the striatum (Table 3), and that of noradrenaline was decreased in the PFC (Figure 3), striatum, hippocampus, midbrain and cerebellum (Table 4).

**Table 1:** Amounts of dopamine and its metabolites (pg mg^-1 ^protein) in each part of the brain.

Brain region	Group	Content (pg mg^-1 ^protein)			
		
		Dopamine	DOPAC	3-MT	HVA
Striatum	Control	270999 ± 30968	20710 ± 2573	21841 ± 3409	32479 ± 3492
	
	Exposed	319714 ± 14364	20197 ± 685	22587 ± 1386	31276 ± 1956

Hippocampus	Control	1520 ± 350	N.D.	714 ± 143	23816 ± 8826
	
	Exposed	951 ± 101	N.D.	449 ± 35	4419 ± 392**

Midbrain	Control	4387 ± 486	2491 ± 191	773 ± 71	3864 ± 225
	
	Exposed	3446 ± 361	1798 ± 110*	555 ± 40*	2847 ± 238*

Cerebellum	Control	202 ± 29	N.D.	N.D.	3225 ± 1152
	
	Exposed	197 ± 46	N.D.	N.D.	4367 ± 1343

Brainstem	Control	17959 ± 646	57513 ± 2294	N.D.	2952 ± 252
	
	Exposed	15775 ± 395*	51445 ± 2244	N.D.	5382 ± 517**

Data are presented as mean ± SEM (*n *= 10 per group). The statistical significance is shown as *P < 0.05, **P < 0.01; ND, not detectable. Abbreviations: DOPAC, 3,4-dihydroxyphenylacetic acid; HVA, homovanillic acid; 3-MT, 3-methoxytyramine.

**Table 2:** Amounts of noradrenaline and its metabolites (pg mg^-1 ^protein) in each part of the brain.

Brain region	Group	Content (pg mg^-1 ^protein)		
		
		Noradrenaline	NM	MHPG
Striatum	Control	1894 ± 310	N.D.	31671 ± 2828
	
	Exposed	1983 ± 278	N.D.	23800 ± 1064*

Hippocampus	Control	9872 ± 1149	691 ± 97	44283 ± 5051
	
	Exposed	10668 ± 625	788 ± 76	30498 ± 1102*

Midbrain	Control	12498 ± 954	410 ± 34	22443 ± 1466
	
	Exposed	12719 ± 633	357 ± 34	14086 ± 675**

Cerebellum	Control	4203 ± 312	615 ± 60	42898 ± 3439
	
	Exposed	4174 ± 262	533 ± 45	28305 ± 1730*

Brainstem	Control	457063 ± 21049	25389 ± 1775	189692 ± 11607
	
	Exposed	430512 ± 18333	20766 ± 1158*	165359 ± 16247

Data are presented as mean ± SEM (*n *= 10 per group). The statistical significance is shown as *P < 0.05, **P < 0.01; ND, not detectable. Abbreviations: MHPG, 4-hydroxy-3-methoxyphenylglycol; NM, normetanephrine.

**Table 3:** Dopamine turnover in each part of the brain.

Brain region	Group	Turnover			
		
		DOPAC/dopamine	3-MT/dopamine	HVA/dopamine	(DOPAC+HVA)/dopamine
Striatum	Control	0.076 ± 0.004	0.077 ± 0.004	0.121 ± 0.004	0.197 ± 0.006
	
	Exposed	0.064 ± 0.002*	0.071 ± 0.003	0.098 ± 0.004*	0.162 ± 0.005**

Hippocampus	Control	--	0.60 ± 0.14	24.66 ± 12.22	--
	
	Exposed	--	0.51 ± 0.06	5.15 ± 0.64	--

Midbrain	Control	0.61 ± 0.05	0.20 ± 0.03	0.99 ± 0.13	1.601 ± 0.165
	
	Exposed	0.55 ± 0.03	0.17 ± 0.01	0.86 ± 0.06	1.404 ± 0.079

Cerebellum	Control	--	--	17.22 ± 5.55	--
	
	Exposed	--	--	39.49 ± 13.83	--

Brainstem	Control	3.22 ± 0.13	--	0.16 ± 0.01	3.38 ± 0.13
	
	Exposed	3.25 ± 0.09	--	0.35 ± 0.04	3.60 ± 0.07

Data are presented as mean ± SEM (*n *= 10 per group). The statistical significance is shown as *P < 0.05, **P < 0.01; ND, not detectable. Abbreviations: DOPAC, 3,4-dihydroxyphenylacetic acid; HVA, homovanillic acid; 3-MT, 3-methoxytyramine.

**Table 4:** Noradrenaline turnover in each part of the brain.

Brain region	Group	Turnover	
		
		NM/noradrenaline	MHPG/noradrenaline
Striatum	Control	--	20.62 ± 3.14
	
	Exposed	--	14.44 ± 2.15

Hippocampus	Control	0.069 ± 0.006	4.59 ± 0.35
	
	Exposed	0.074 ± 0.007	2.97 ± 0.24**

Midbrain	Control	0.034 ± 0.002	1.84 ± 0.11
	
	Exposed	0.028 ± 0.003	1.12 ± 0.06**

Cerebellum	Control	0.145 ± 0.011	10.82 ± 1.30
	
	Exposed	0.131 ± 0.013	6.87 ± 0.36*

Brainstem	Control	0.0568 ± 0.003	0.415 ± 0.015
	
	Exposed	0.0487 ± 0.003	0.380 ± 0.030

Data are presented as mean ± SEM (*n *= 10 per group). The statistical significance is shown as *P < 0.05, **P < 0.01; ND, not detectable. Abbreviations: MHPG, 4-hydroxy-3-methoxyphenylglycol; NM, normetanephrine.

### Serum corticosterone

The levels of serum corticosterone were not different between the exposure group and the control group (data not shown).

## Discussion

This is the first study that has demonstrated the effects of *in utero *exposure to a low concentration of diesel exhaust (0.171 mg DEP/m^3^) on locomotor activity and monoamine level in brain tissue. In the present study, we used only male fetuses and pups for analysis because the prevalence of some psychiatric disorders in childhood, such as autism and attention deficit hyperactivity disorder, is higher in men than in women. The results of this study demonstrated that SLA in a new environment and in the home cage environment was decreased and monoaminergic neurochemistry in several regions of the brain was altered in the exposure group. SLA in the home cage environment was particularly decreased when the lights turned on. The alteration of decreased SLA and dopamine turnover in the striatum was similar to a finding in an earlier study that examined the effects of prenatal exposure to a relatively higher concentration of diesel exhaust (1.0 mg DEP/m^3^) [17]. The present study showed alteration of the monoamine metabolism in other regions of the CNS, especially the PFC, even by exposure to a lower concentration of diesel exhaust.

It has been reported that diesel exhaust and DEPs contain estrogenic and antiestrogenic compounds and possess endocrine-disrupting activity [20-23]. In the present study, the number of female pups of the exposure group was less than half of that of the control group. It may indicate that embryogenesis of female fetuses was affected by the activity of DEPs. Tsukue *et al*. [24] examined the effects of exposure to diesel exhaust during the perinatal period on sexual differentiation-related gene expression of the brain. Expression levels of *estrogen receptor (ER) α *and *ER β mRNA *were increased in the cerebrum of newborns in the exposure group as well as mRNA for *CYP1A1 *and *HO-1*. The results indicate that prenatal exposure to diesel exhaust during the critical period of sexual differentiation of the brain may affect endocrine function. Estrogen has many important roles in the brain, including brain development, neuroprotection such as inhibition of apoptosis and synaptogenesis, and functions of the monoaminergic systems [25], the effects of perinatal exposure to diesel exhaust on CNS have been focused on. There is a report that shows serum level of estradiol is increased in male rats exposed to diesel exhaust after birth [26]. However, whether the serum estradiol level is affected by prenatal exposure to diesel exhaust remain unknown. Further investigation is required to clarify the mechanisms of the effects of diesel exhaust exposure on the levels of estradiol and CNS of offspring.

When pregnant mice were exposed to diesel exhaust, cytoplasmic granules of granular perithelial cells contained ultrafine DEP-like particles and the apoptosis of endothelial cells and stenosis of some capillaries were observed [16]. Furthermore, caspase-3 positive cells were observed in the cerebral cortex and in the hippocampus of the newborn [27]. These observations suggest that exposure of pregnant mice to diesel exhaust might carry a risk of cellular atrophy and might affect development of the fetal brain. A review by Herlenius and Lagercrantz [28] indicated that stimulation or insult at critical phases of development of the nervous system could result in long-term changes in organismal structure and function. Perinatal exposure to environmental contaminants, including DEPs, that have hormone-like activity [20-23] and can generate reactive oxygen species [29], might be able to disturb the timetable of expression of neurotransmitters and neuromodulators and their receptors, evoking permanent changes in cellular proliferation and differentiation and in growth, leading to behavioural and neurophysiologic abnormalities. The details of the mechanism how DEPs affect fetus and damage CNS of offspring remain unclear. However, when gold nanoparticle (1.4 - 18 nm) is intravenously injected into female rats, inversely size-dependent uptake in the placenta and translocation into the fetus were found [30]. Nanosized fraction of DEPs may majorly contribute to adverse effects on CNS of offspring. The toxicity of DEP for dopamine neurons was shown in an *in vitro *study [12] and the toxicity for CNS *in vivo *should be further investigated. The present study showed that daily SLA decreased in the exposure group compared to that in the control group when the mice were put into a new environment. This alteration is similar to that of ovariectomized mice treated with estradiol [31,32], suggesting that it may be caused by estrogenic activity of diesel exhaust. The dopamine and noradrenaline systems in the PFC have an important role in the control of locomotor activity. Destruction of mesocortical and dopamine projections in rats results in increased motor activity [33-36], suggesting that one of the roles of dopamine in the PFC is to suppress locomotor activity. In the present study, the data showed that dopamine and its metabolites were increased in the PFC of the exposure group and the basal stress level was not altered by diesel exhaust exposure because the levels of serum corticosterone were not different between groups. The dopamine and noradrenaline systems in the PFC are responsive to various stressors [37,38]. The response of the dopamine system is independent of the pituitary-adrenocortical axis [39]. The cause of decreased SLA in mice exposed prenatally to diesel exhaust may be a transiently facilitated release of dopamine as a result of exposure to novel stimuli rather than alteration of the level of basal stress. Our unpublished data showed that the effects of diesel exhaust exposure on CNS of offspring are reduced by removing the DEPs from the exhaust with a filter. It suggests that particulate component of diesel exhaust contribute to the effects of maternal exposure to diesel exhaust on CNS.

## Conclusions

The exposure to low concentrations of diesel exhaust *in utero *decreased spontaneous locomotor activity and altered monoaminergic neurochemistry in several regions of the brain in male mice. However, the mechanism connecting the behavioural and neurochemical alterations remains unclear. We cannot rule out an indirect effect of diesel exhaust exposure via the mother's behaviour toward the pups and how this in turn altered SLA and monoamine metabolism in the offspring since this was not investigated. Further investigations are needed to clarify the critical factor for the effects on offspring. Since current observations are done at a particle mass concentration of diesel exhaust close to the environmental quality standard of daily-averaged level of suspended particulate matter (SPM) in Japan, these finding warrant revisiting of present air quality standards for particulate matter.

## List of abbreviations used

CNS: central nervous system; DEP: diesel exhaust particle; DOPAC: 3,4-dihydroxyphenylacetic acid; GD: gestational day; HPLC: High-performance liquid chromatography; HVA: homovanillic acid; MHPG: 4-hydroxy-3-methoxyphenylglycol hemipiperazinium; 3-MT: 3-methoxytyramine hydrochloride; NM: normetanephrine hydrochloride; PFC: prefrontal cortex; PM: particulate matter; PND: postnatal day; SLA: spontaneous locomotor activity; SPM: suspended particulate matter.

## Competing interests

The authors declare that they have no competing interests.

## Authors' contributions

TS, SO, MI, HS and TO were substantially involved in conducting the experiments. TS and MU was involved in data analyses and in drafting the manuscript. TU and IS conducted and controlled the diesel exhaust exposure. KT is the main project leader and conceived the overall research idea. All authors read and approved the final manuscript.
